# Effects of Serial Polydrug Use on the Rewarding and Aversive Effects of the Novel Synthetic Cathinone Eutylone

**DOI:** 10.3390/brainsci13091294

**Published:** 2023-09-07

**Authors:** Hayley N. Manke, Samuel S. Nunn, Agnieszka Sulima, Kenner C. Rice, Anthony L. Riley

**Affiliations:** 1 Psychopharmacology Laboratory, Center for Neuroscience and Behavior, Department of Neuroscience, American University, 4400 Massachusetts Ave, NW, Washington, DC 20016, USA; hm2522a@american.edu (H.N.M.);; 2 Drug Design and Synthesis Section, National Institute on Drug Abuse (NIDA), National Institute on Alcohol Abuse and Alcoholism (NIAAA), Bethesda, MD 20892, USAkennerr@nida.nih.gov (K.C.R.)

**Keywords:** taste avoidance, place preference, eutylone, MDMA, cocaine, drug history

## Abstract

**Highlights:**

- Eutylone induced dose-dependent CTA and CPP.
- MDMA pre-exposure attenuated eutylone-induced CTA, but it did not impact eutylone-induced CPP.
- Cocaine pre-exposure had no significant effect on eutylone-induced CTA or CPP.
- The weak effects of eutylone history on cocaine and MDMA may be a function of eutylone’s specific hybrid neurochemical actions on DA and 5-HT.

**Abstract:**

Background: As individual synthetic cathinones become scheduled and regulated by the Drug Enforcement Administration (DEA), new ones regularly are produced and distributed. One such compound is eutylone, a novel third-generation synthetic cathinone whose affective properties (and abuse potential) are largely unknown. The following experiments begin to characterize these effects and how they may be impacted by drug history (a factor affecting reward/aversion for other drugs of abuse). Methods: Eutylone was assessed for its ability to induce conditioned taste avoidance (CTA; aversive effect) and conditioned place preference (CPP; rewarding effect) and their relationship (Experiment 1). Following this, the effects of exposure to cocaine or 3,4-methylenedioxymethamphetamine [MDMA] on eutylone’s affective properties were investigated (Experiment 2). Results: Eutylone produced dose-dependent CTA and CPP (Experiment 1), and these endpoints were unrelated. Pre-exposure to cocaine and MDMA differentially impacted taste avoidance induced by eutylone (MDMA > cocaine) and did not impact eutylone-induced place preference. Conclusions: These data indicate that eutylone, like other synthetic cathinones, has co-occurring, independent rewarding and aversive effects that may contribute to its abuse potential and that these effects are differentially impacted by drug history. Although these studies begin the characterization of eutylone, future studies should examine the impact of other factors on eutylone’s affective properties and its eventual reinforcing effects (i.e., intravenous self-administration [IVSA]) to predict its use and abuse liability.

## 1. Introduction

The affective properties of drugs known to contribute to drug use and abuse are impacted by a host of subject (e.g., age, sex, strain) and experiential (e.g., dose, route, drug combinations) factors, one of which is drug history (i.e., serial interactions). In relation to drug history, exposure to a drug prior to conditioning attenuates and sensitizes acquisition of taste avoidance and place preference conditioning, respectively (for reviews and various associative and non-associative interpretations, see [1,2,3,4]). Given that the relative balance of a drug’s aversive and rewarding effects has been reported to mediate the likelihood of its use (see [5,6,7,8,9]), understanding the effects of drug history may be important in predicting its abuse liability.

This potential impact of drug history is especially relevant to the use and abuse of synthetic cathinones (derivatives of the naturally occurring stimulant cathinone; see [10,11]). Several studies report that synthetic cathinone users engage in serial polydrug use (i.e., the use of multiple drugs across multiple sessions; see [12,13,14]). For example, Smith and Stoops [15] noted that users of synthetic cathinones significantly surpass those reporting no lifetime synthetic cathinone use in the rates of lifetime and past year use of several drugs including club drugs/hallucinogens (76.6% vs. 64.4%) and barbiturates (58.2% vs. 28.2%), among others. Further, Fernández-Calderón et al. [16] surveyed individuals attending electronic dance music (EDM) parties in New York City to identify patterns of use of illicit drugs among this population and observed that 19.2% of attendees reported engaging in extensive polydrug use in the past year (the mean number of drugs being 6.4). Of those reporting polydrug use in the past year, 5.3% of these individuals reported past-year synthetic cathinone use as well (see also [17,18]).

Synthetic cathinones are a constantly evolving issue as established compounds are scheduled [19,20,21] and new ones are introduced in their place [22,23,24]. One such compound is eutylone, a novel third-generation synthetic cathinone that has recently emerged in global recreational drug markets ([25,26,27]; see also [28,29,30,31]). Interestingly, recent reports have indicated that the use of stimulant drugs such as MDMA, synthetic cathinones (including eutylone) and cocaine account for a majority of drug use at music festivals and clubs [32,33,34,35]. The likelihood of serial use is further increased by the fact that stimulants such as cocaine and MDMA are often adulterated with (or substituted for) structurally related analogues such as synthetic cathinones [36,37]. For example, West et al. [37] analyzed trace residues of discarded drug packing samples from large public events, e.g., EDM festivals, and found that cocaine and MDMA were some of the most popular compounds along with eutylone, ethylone and N-ethylpentylone. Further, all three of these synthetic cathinones were found in combination with MDMA, while eutylone was observed with cocaine.

Eutylone is especially interesting in that it is described as “hybrid” in nature, resulting from chemical modifications to its parent compounds (e.g., methylone; for a discussion, see [38,39]). While the majority of synthetic cathinones act as monoamine reuptake inhibitors or substrate releasers, eutylone acts as a hybrid with reuptake inhibitor properties at DAT and NET and substrate release activity at SERT [40]. The reuptake inhibition of eutylone (and other synthetic cathinones) at DAT is consistent with the pharmacological action of cocaine [41,42], while its action at SERT is akin to that of MDMA [43,44,45], suggesting that eutylone may have psychostimulant effects and abuse vulnerability comparable to these and related compounds.

To begin to address the possible serial interactions of eutylone with other drugs that share, in part, its neurochemical activity, the present study examined the effects of cocaine or MDMA pre-exposure on the aversive and rewarding effects of eutylone in mice in a combined CTA/CPP design, which allows for a concurrent assessment of both affective properties in the same subject (for examples, see [46,47,48,49,50]). In this design, animals are given access to a novel taste, injected with the drug and then placed on one side of a place preference chamber, allowing for an assessment of the acquisition of a taste avoidance (index of the drug’s aversive effects) and a place preference (index of the drug’s rewarding effects). This design facilitates concurrent assessments of these properties given the ability of animals to selectively associate the taste with the aversive effects of the drug and environmental cues with the drug’s rewarding effects (for discussions of selective associations shaped by evolutionary pressures, see [51,52,53,54]). This question was addressed via two studies. In Experiment 1, male C57BL/6 mice were conditioned with a range of doses of eutylone (0, 1, 3.2, 10 or 32 mg/kg) to assess its ability to induce taste avoidance and place preference, indices of its aversive and rewarding effects, respectively). Following determinations of doses of eutylone effective in inducing these effects, additional male C57BL/6 mice, in Experiment 2, were exposed to 32 mg/kg (MDMA) or 3.2 mg/kg (cocaine) every 4th day (for a total of five injections) prior to CTA and CPP training with eutylone. As noted, such drug pre-exposure has been reported to impact both the rewarding and aversive effects of drugs (as measured in place preference and taste avoidance conditioning, respectively), especially when the drugs share similar neurochemical actions [55,56,57,58,59]. Given that eutylone is a hybrid compound with both DA and 5-HT activity (see above), it is expected that both cocaine (DA) and MDMA (5-HT) pre-exposure may impact its affective properties.

## 2. Experiment 1

### 2.1. General Methods

#### 2.1.1. Subjects

The subjects were male (*n* = 40) experimentally naïve C57BL/6 mice bred within the American University animal research facility. Subjects matured undisturbed until the start of testing. Starting between post-natal day (PND) 56–84 (8–12 weeks of age), animals were weighed daily for at least 7 days to index health status and decrease handling stress during experimental procedures. Subjects were run in two replicates, each of which contained an equal number of animals (*n* = 20) with all groups represented (see below). At the outset, subjects weighed between 20.3 g and 29.2 g (replicate 1: mean = 25.1; SEM = 0.392; replicate 2: mean = 26.1; SEM = 0.404). In both replicates, five groups of subjects (*n* = 4 per group) were examined daily. The procedures utilized in the present work adhered to the Guidelines for the Care and Use of Laboratory Animals [60] and the Guidelines for the Care and Use of Mammals in Neuroscience and Behavioral Research [61] and were approved by the Institutional Animal Care and Use Committee at American University.

#### 2.1.2. Apparatus

Subjects were housed four per group in OptiMouse cages (13.5 × 11.5 × 6.1; 75 sq. in). The animal housing room was kept on a 12 h light/dark cycle (0800–2000 h) and at 23 °C. All training and testing procedures occurred during the lights-on phase of the light cycle. Unless stated otherwise, food and water were available ad libitum. During CTA training and testing, animals were transferred to separate individual OptiMouse cages for fluid presentation in which graduated Nalgene tubes were placed on the side of the cage for fluid consumption. For CPP assessments, subjects were transferred to one of eight identical three-chambered CPP systems (68.5 × 21 × 34.5 cm; San Diego Instruments Place Preference System, San Diego, CA, USA). Each CPP apparatus contained three distinct areas, each of which was equipped with a photobeam array that recorded time spent in specific locations within the apparatus. On the left side chamber of the apparatus (28 × 21 × 34.5 cm), there were white walls and white metal diamond-plate flooring, and on the right side (28 × 21 × 34.5 cm), there were black walls and black plastic hair-cell textured flooring. The middle chamber (14 × 21 × 34.5 cm), which was not used for testing, contained grey walls and metal grid flooring that consisted of metal rods placed approximately 1 cm apart. All chambers (and the room in which they were contained) were unlit, and a white noise generator was used to mask background noise that could interfere with conditioning.

#### 2.1.3. Drugs and Solutions

Racemic eutylone (β-keto-1,3-benzodioxolyl-N-ethylbutanamine) was synthesized and generously provided by the Drug Design and Synthesis Section, MTMDB, NIDA and NIAAA and by the NIDA Drug Supply program. Eutylone was dissolved in isotonic saline (0.9%) and injected intraperitoneally (IP) at 1, 3.2, 10 and 32 mg/kg. Concentration was held constant, and as such, injection volume was dependent on the eutylone dose group to which subjects were assigned. Isotonic saline (vehicle) was administered to control subjects equivolume to the highest dose of eutylone. Each drug (and vehicle) solution was prepared daily and passed through a 0.2-um filter prior to injection to remove any potential particulates. Saccharin (Sodium Saccharin, Acros Organics) was prepared as a 1 g/L (0.1%) solution in tap water.

#### 2.1.4. Procedure

##### Combined CTA/CPP Design

1. Water Habituation

**Figure 1:**
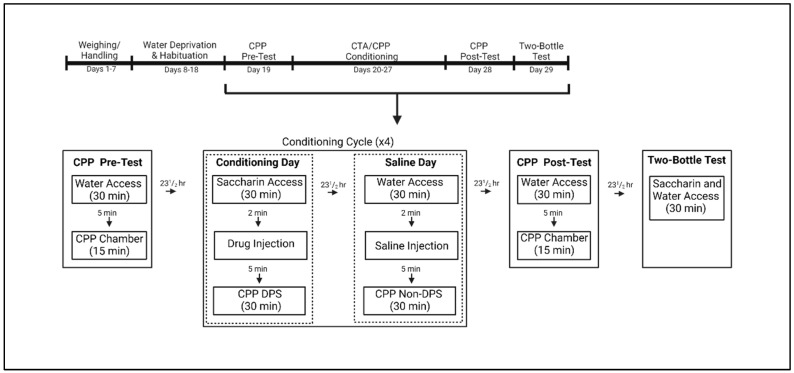
Experimental Timeline for subjects undergoing the combined conditioned taste avoidance (CTA)/conditioned place preference (CPP) procedure. On drug days, animals were given saccharin followed by a drug injection and placed on the drug–paired side (DPS). On saline days, animals were given water access followed by a vehicle (saline) injection and placed on the non–drug–paired side (Non–DPS). Created with BioRender.

Beginning 24 h before experimental procedures, subjects were deprived of water and the next day were given 30 min access to tap water in the individual plastic testing cages. Animals were then returned to their home cages immediately following water access, and each testing cage was cleaned using Sani-Plex 128M, a one-step disinfectant germicidal detergent, between subjects. The limited fluid access procedure was used to induce water consumption during subsequent testing sessions and was repeated until animals approached the drinking tube within 2 s and average water consumption did not change by >0.15 mL for 3 consecutive days. Water was presented in graduated 50 mL Nalgene tubes that were marked in increments of 0.5 mL, and intake was evaluated by the difference between pre- and post-consumption water volumes (see Figure 1, Experimental Timeline).

2. CPP Pre-Test

Following stabilization of water consumption, subjects were given 30 min access to water in the test cages before being placed in the middle grey chamber of the CPP chamber and allowed to freely explore for 15 min. Animals were immediately returned to their home cages following the session. To determine if there were initial side preferences for each replicate, a paired samples t-test on the absolute time spent on the white side vs. absolute time spent on the black side during the 15 min testing period was conducted and indicated an unbiased apparatus (replicate 1: t = −1.463, *p* = 0.160; replicate 2: t = −1.446, *p* = 0.164). Although statistically unbiased, there were three animals that spent more than 65% of the 15 min testing time on one side of the apparatus during the Pre-Test suggestive of a strong natural bias and therefore were excluded from the statistical analysis of place preference and taste avoidance conditioning (although still run in the behavioral assessments). As mentioned above, the middle chamber was not used in conditioning (i.e., not paired with drug or saline) and, as such, was not used in the calculation of side preferences. Between animals, each chamber was thoroughly cleaned using Sani-Plex 128M.

3. CTA/CPP Conditioning

On the first day of conditioning (Day 1), subjects were placed in their individual test cages in groups of eight and given 30 min access to a novel saccharin solution. After saccharin access, subjects were assigned to conditioning groups such that saccharin consumption among groups was comparable. Subjects were assigned to one of five drug groups and subsequently injected IP with either saline or 1, 3.2, 10 or 32 mg/kg of racemic eutylone. Concentration was held constant, and as such, injection volume was dependent on the eutylone dose to which subjects were assigned. The dose range used in the present study is based on the work of Glatfelter et al. [40] in which doses of 0, 3, 10 and 30 mg/kg of eutylone administered subcutaneously were used to induce locomotion in male C57BL/6 mice. This resulted in a total of five groups, i.e., Groups E0, E1, E3.2, E10 and E32, where the letter indicates eutylone and the number indicates drug dose (*n* = 7–8 per group). A power analysis indicated that *n* ≥ 7 is appropriate to detect significant differences with the anticipated effect sizes and with α = 0.05 and power (1 − β) = 0.8. Following drug injections, subjects in each drug group were assigned in a counterbalanced fashion to their preferred or non-preferred side (i.e., half placed on their preferred and half placed on their non-preferred) as defined in the Pre-Test as the side in which the mouse spent the most time for 30 min. Subjects were then returned to their home cages, and the test cages and place preference chambers were sanitized prior to the next set of animals. On the next day (Day 2), they were given 30 min access to water in the test cages, injected with vehicle and placed on the opposite side of the place preference chamber for 30 min. This two-day cycle was repeated for a total of four cycles.

4. CPP Post-Test and CTA Two-Bottle Test

Subsequent to four conditioning cycles (Day 9 of testing), subjects were given 30 min access to tap water, placed in the center chamber and allowed to freely explore all three chambers for 15 min. Time spent on the DPS and Non-DPS was recorded to determine the percentage of time spent on each side. Following the CPP Post-Test (Day 10), animals were placed in plastic test cages and given 30 min access to both saccharin and tap water in a two-bottle avoidance test with no subsequent injections. The two-bottle test was used in the present study given its increased sensitivity that may detect effects not seen during CTA acquisition (see [62,63,64,65]). In this test, animals are not forced to choose between consumption and avoidance as they are given a choice between the drug-paired solution and a neutral or safe fluid (e.g., water). During the two-bottle test, one bottle was offered (saccharin or water) and once sampled by the animal, it was removed, and the second bottle was presented. After both bottles were sampled, they were placed simultaneously on their respective sides of the cage for 30 min and then consumption of both solutions was measured. Both the order of presentation and side placement were counterbalanced across animals. Once testing concluded, animals were returned to their home cages and given ad libitum water access. The percentage of saccharin consumed was calculated by dividing saccharin consumption by total fluid consumption (volume of saccharin + volume of water) and multiplied by 100.

5. Statistical Analysis

The percentage change in saccharin consumption (Trial 1 − Trial 4/Trial 1 × 100 = percentage change across CTA) during CTA acquisition was analyzed using a one-way ANOVA with the between-subjects factor of Dose (0, 1, 3.2, 10, 32 mg/kg). The percentage of saccharin consumed on the two-bottle avoidance test and percentage time on the DPS on the CPP Post-Test were analyzed using a one-way ANOVA with the same between-subjects factor. In the case of a significant interaction, multivariate analyses were assessed followed by Bonferroni-adjusted multiple comparisons.

The relationship between the percentage of saccharin consumed on the two-bottle test and the percentage of time spent on the DPS on the CPP Post-Test for each dose was determined for mice injected with eutylone using Pearson correlation coefficients. Statistical significance was set to *p* < 0.05.

### 2.2. Results

#### 2.2.1. Conditioned Taste Avoidance

**Figure 2:**
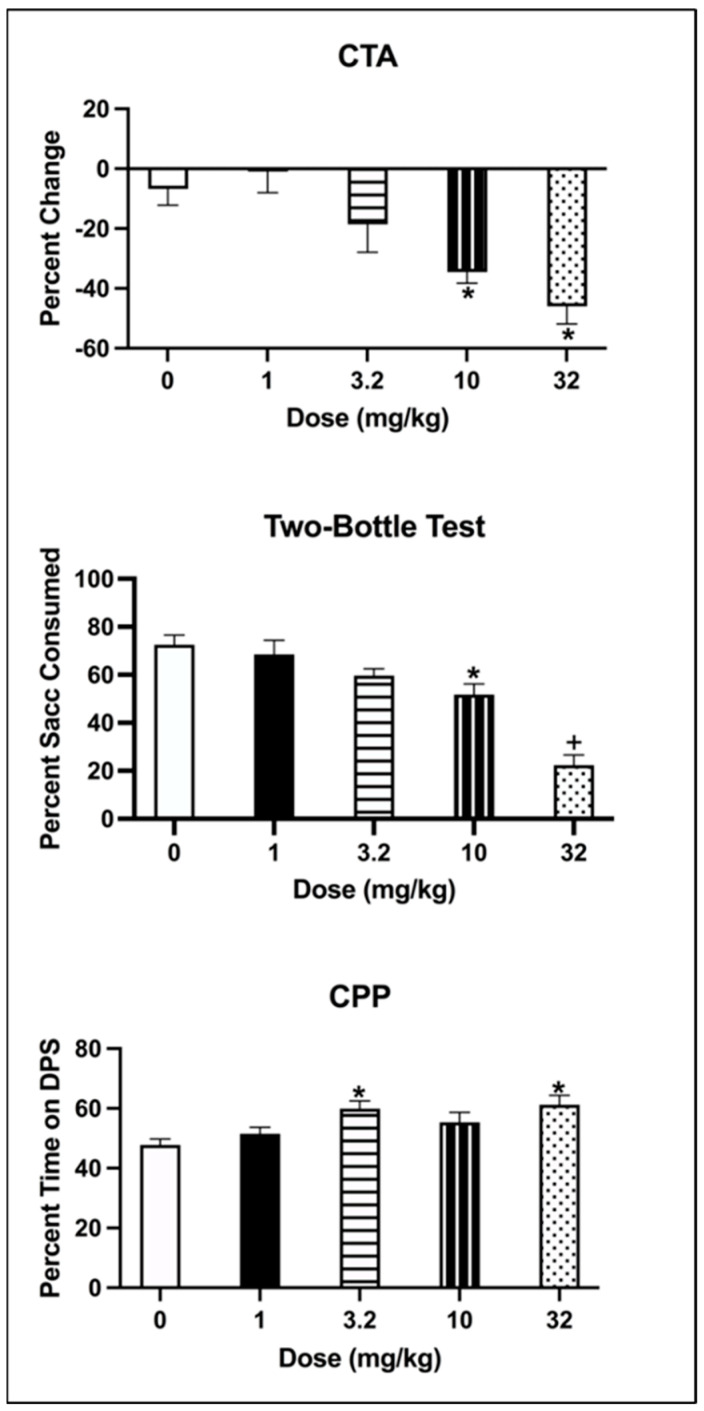
(**Top**): Mean (±SEM) percentage change in saccharin consumption (ml) for subjects injected with vehicle (0) or eutylone at 1, 3.2, 10 or 32 mg/kg. * 10 and 32 mg/kg significantly differed from vehicle and 1 mg/kg; (**Middle**): Mean (±SEM) percentage saccharin consumed on the two-bottle test for subjects injected with vehicle (0) or 1, 3.2, 10 or 32 mg/kg eutylone. * 10 significantly differed from vehicle. ^+^ 32 mg/kg significantly differed from vehicle, 1, 3.2 and 10 mg/kg; (**Bottom**): Mean (±SEM) percentage time on the drug-paired side (DPS) on the CPP Post–Test for subjects injected with vehicle (0) or 1, 3.2, 10 or 32 mg/kg eutylone. * 3.2 and 32 mg/kg significantly differed from vehicle.

The one-way ANOVA on the percentage change in saccharin consumption (from Trial 1 to Trial 4) showed a significant effect of Dose [F(4, 35 = 8.272, *p* < 0.001] (see Figure 2; top panel). Animals injected with 1 and 3.2 mg/kg of eutylone did not differ from the controls (all ps > 0.05), while those injected with 10 or 32 mg/kg of eutylone had a significantly greater percentage decrease than the controls (all ps < 0.05). Further, animals injected with 10 or 32 mg/kg had significantly greater decreases in saccharin than those treated with 1 mg/kg (all ps < 0.05).

#### 2.2.2. Two-Bottle Test

The one-way ANOVA on the percentage of saccharin consumed on the two-bottle test indicated that there was a main effect of Dose [F(4, 35) = 20.401, *p* < 0.001] (see Figure 2: middle panel). While subjects conditioned with 1 or 3.2 mg/kg did not differ from the controls (all ps > 0.05), those injected with 10 or 32 mg/kg consumed a significantly lower percentage of saccharin than those injected with the vehicle (all ps < 0.05). Subjects injected with 32 mg/kg also had a significantly lower percentage of saccharin consumed than subjects injected with 1, 3.2 or 10 mg/kg (all ps < 0.05).

#### 2.2.3. Conditioned Place Preference

The one-way ANOVA on the percentage of time spent on the drug-paired side (DPS) indicated that there a significant main effect of Dose [F(4, 32 = 4.509, *p* = 0.005]. Subjects conditioned with 3.2 and 32 mg/kg of eutylone spent a significantly greater percentage of time on the DPS than vehicle animals (all ps < 0.05) (see Figure 2, bottom panel). Subjects conditioned with 1 or 10 mg/kg did not differ from the controls (all ps > 0.05).

#### 2.2.4. CTA/CPP Relationship

**Figure 3:**
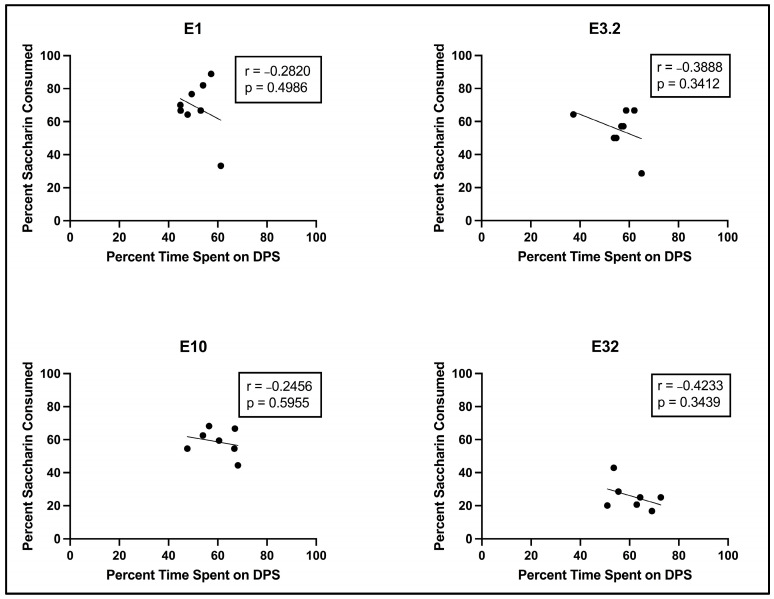
Scatterplots (with best line of fit), displaying the relationship between percentage of saccharin consumed on the two-bottle test and percentage of time spent on the DPS during the CPP Post–Test for subjects injected with eutylone.

Analysis of the relationship between percentage of saccharin consumed on the two-bottle test and percentage of time spent on the DPS during the CPP Post-Test revealed no significant relationship at any dose (see Figure 3 below).

### 2.3. Discussion

Eutylone induced significant dose-dependent CTA (at 10 and 32 mg/kg) and CPP (at 3.2 and 32 mg/kg), and these effects were not correlated indicating they are independent and dissociable. The fact that eutylone was effective in induced taste avoidance and place preference conditioning is consistent with prior work with rats (although avoidance was stronger in rats than mice, effects consistent with other work in which mice and rats have been compared; see [48,66,67,68,69]; see below). Although there are few studies that have examined the relationship between taste avoidance and place preference, the data that have been collected appear to be mixed. For example, Turenne et al. [70] did not report a relationship between morphine-induced taste avoidance and place preference in a serial taste/place conditioning procedure (e.g., CTA was conducted followed by CPP conditioning); however, they did find a significant positive relationship between amphetamine-induced CTA and CPP at the highest dose administered. Verendeev and Riley [71] also reported a relationship with animals conditioned with the highest dose of amphetamine (but not lower doses) in the combined CTA/CPP design, although the relationship under their experimental parameters was opposite to that described by Turenne et al., i.e., subjects that showed greater decreases in saccharin consumption were less likely to display a place preference. Similar to Turenne et al., Verendeev and Riley reported no relationship with morphine at any dose tested. In relation to the synthetic cathinones, King et al. [72] found a significant inverse relationship for females conditioned with 1.8 mg/kg of MDPV but not at any other dose (and no relationships with males at any dose). Further, we recently demonstrated that for eutylone’s parent compound methylone, there was no significant relationship when collapsed across dose (r = 0.1853; *p* = 0.2230), but when examined by dose, there was a significant inverse relationship at the 5.6 mg/kg dose, i.e., as CPP increased, CTAs decreased (r = 0.6324; *p* = 0.0086) [48]. Such a relationship is similar to that reported by King et al. and Verendeev and Riley. Despite the few occasions in which a significant relationship is reported, the general consensus (i.e., in roughly 75–80% of cases) is that there is no significant correlation between CTA and CPP, supporting the notion that taste avoidance and place preference are independent, co-occurring stimulus properties of drugs. In cases where significant correlations are seen, this may be a function of chance occurrences when conducting multiple comparisons. Importantly, such assessments must be done with the synthetic cathinones (and other drugs) to determine the outcome of such analyses and under what conditions significant relationships occur given that it may provide insight into the nature of reward and aversion for drugs of abuse (for a discussion, see [71]).

The rewarding and aversive effects and their dissociation reported above are consistent with other drugs of abuse (for a review, see [7,9]), including synthetic cathinones [48,69,72]. Given the data demonstrating effective doses of eutylone in taste and place conditioning, Experiment 2 assessed the potential interaction of compounds neurochemically related to eutylone. Specifically, mice were exposed to either cocaine or MDMA prior to taste avoidance and place preference conditioning with eutylone to examine potential changes in its affective properties.

## 3. Experiment 2

### 3.1. General Methods

Unless otherwise specified, the strain of animals, housing conditions and the specific training procedures (CTA/CPP) utilized here were identical to those described for Experiment 1 (see above).

#### 3.1.1. Subjects

The subjects were male (*n* = 96) experimentally naïve C57BL/6 mice. Starting between post-natal days (PND) 56–84 (8–12 weeks of age), animals were weighed daily for a minimum of 6 days to index health status and reduce handling stress during the subsequent experimental procedures. They were run in two replicates, each of which had an equal number of animals (*n* = 48 total/replication) and represented all drug groups. For each replicate, six groups of subjects (*n* = 8 per group) were assessed daily. Subjects weighed between 21.6 g and 29 g (replicate 1: mean = 25.8; SEM = 0.197; replicate 2: mean = 24.2; SEM = 0.198) at the start of experimental procedures.

#### 3.1.2. Drugs and Solutions

Cocaine hydrochloride and MDMA hydrochloride were synthesized and generously provided by the National Institute on Drug Abuse (NIDA) Drug Supply Program and were dissolved in isotonic saline (0.9%) and injected subcutaneously (SC) at 32 and 3.2 mg/kg, respectively. These doses were based on prior work reporting that each is effective in inducing taste avoidance (for cocaine, see [73,74,75,76]; for MDMA, see [58,77,78,79]) and that both attenuate taste avoidance following their pre-exposure (for cocaine, see [80,81]; for MDMA, see [77]). Concentration for the cocaine solution was 2 mg/mL and for MDMA was 0.5 mg/mL. Controls were administered equivolume saline SC during pre-exposure.

Racemic eutylone was synthesized and generously provided by the Drug Design and Synthesis Section, MTMDB, NIDA and NIAAA and by the NIDA Drug Supply program. Eutylone was dissolved in isotonic saline (0.9%) and injected IP at 3.2, 10 and 32 mg/kg. Concentration was held constant, and as such, injection volume was dependent on the eutylone dose group to which subjects were assigned. This dose range is based on the results of Experiment 1. Isotonic saline (vehicle) was administered to controls, equivolume to that administered for the high dose eutylone group.

#### 3.1.3. Procedure

##### Water Habituation and Cocaine/MDMA Pre-Exposure

**Figure 4:**
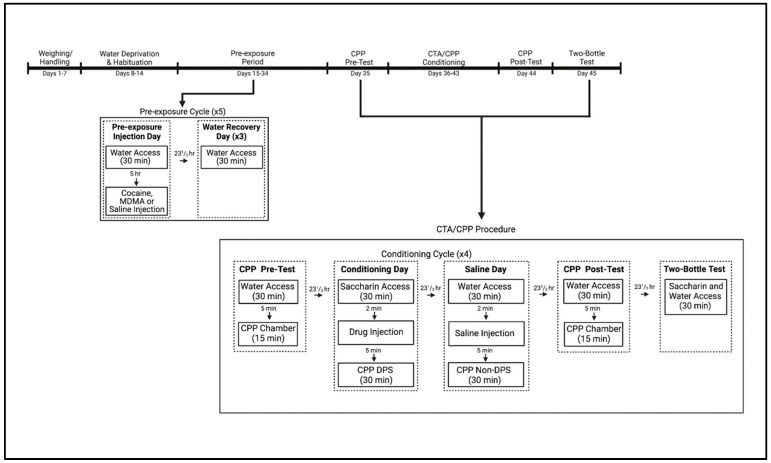
Experimental Timeline for subjects exposed to vehicle, cocaine or MDMA every 4th day (for a total of 5 injections) prior to undergoing a combined conditioned taste avoidance (CTA)/conditioned place preference (CPP) procedure. On conditioning days, animals were given saccharin followed by a drug injection and placed on the drug–paired side (DPS). On saline days, animals were given water access followed by a saline injection and placed on the non–drug–paired side (non–DPS). Created with BioRender.

At the beginning of experimental procedures, mice were deprived of water for 24 h before being transferred to individual plastic cages and given 30 min access to tap water delivered in 50 mL Nalgene tubes. The mice were given 6 days to habituate to the limited fluid-access procedure. On the 7th day of limited access, mice were matched on water consumption and assigned to three groups (*n* = 32 for each group) such that average consumption was comparable among groups. Approximately 5 h after fluid access, mice were removed from their home cages and transferred to a separate room where they were injected SC with vehicle, cocaine or MDMA before being returned to their home cages. For the following 3 days, mice received 30 min water access but no injections. This procedure of vehicle, cocaine or MDMA exposure followed by 3 recovery days was repeated for a total of five cycles over the course of 20 days (see Figure 4; Experimental Timeline; for other work utilizing this procedure, see [58,77,81,82]).

##### Combined CTA/CPP Design

1. CPP Pre-Test

Animals were assessed for initial side preferences (see Experiment 1). Paired samples t-test on the absolute time spent on the white side vs. absolute time spent on the black side during the 15 min testing period indicated a biased apparatus (t = 2.582, *p* = 0.013) for replicate 1 and unbiased apparatus (t = −0.881, *p* = 0.383) for replicate 2. To maintain comparable experimental conditions, both replicates were run using a biased procedure (see below). Additionally, four animals spent more than 65% of the 15 min testing time on one side of the conditioning chamber during the Pre-Test and were excluded from the statistical analysis (although still run in all behavioral assessments).

2. CTA/CPP Conditioning

During conditioning, subjects from each pre-exposure condition were assigned to one of four conditioning groups such that saccharin consumption among groups was comparable. Immediately following saccharin access, they were injected IP with the vehicle or 3.2, 10 or 32 mg/kg of racemic eutylone. This resulted in a total of 12 experimental groups, i.e., V0, V3.2, V10, V32, C0, C3.2, C10, C32, M0, M3.2, M10 and M32 where the first letter indicates the pre-exposure condition (vehicle, cocaine or MDMA) and the number indicates the dose of eutylone (0, 3.2, 10 or 32 mg/kg) (*n* = 7–8 for each group). A biased training procedure was employed during conditioning such that each animal was placed on its non-preferred side following drug injections. Chambers were sanitized between animals.

3. CPP Post-Test and CTA Two-Bottle Test

The CPP Post-Test and Two-Bottle Test were conducted as described in Experiment 1.

#### 3.1.4. Statistical Analysis

##### Cocaine

To assess whether cocaine pre-exposure affected body weight and fluid consumption, a 2 × 5 mixed model ANOVA with the between-subjects factor of Pre-exposure Drug and the within-subjects factor of Injection Day was run separately for each measure.

Percentage change in saccharin consumption (from Trial 1 to Trial 4) during CTA acquisition was analyzed separately for each dose using a two-way ANOVA with the between-subjects factors of Pre-Exposure Drug (Vehicle or Cocaine) and Conditioning Drug (vehicle or eutylone). Percentage saccharin consumed on the two-bottle avoidance test and percentage time on the DPS on the CPP Post-Test were analyzed for each dose using a two-way ANOVA with the same between-subjects factors. In the case of a significant interaction, univariate analyses were assessed followed by Bonferroni-adjusted multiple comparisons.

##### MDMA

Effects of MDMA on body weight and water consumption during pre-exposure were assessed as described for cocaine. Percentage change in saccharin consumption (from Trials 1–Trial 4) during CTA acquisition as well as percentage saccharin consumed on the two-bottle test and percentage time on the DPS on the CPP Post-Test for MDMA-pre-exposed subjects were analyzed as described above.

Statistical significance was set to *p* < 0.05.

### 3.2. Results

#### 3.2.1. Cocaine Pre-Exposure

##### Body Weight and Water Consumption during Pre-Exposure

**Figure 5:**
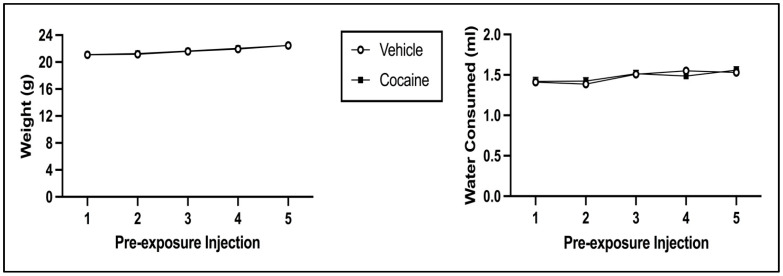
Mean (±SEM) body weight (**left**) and water consumption (**right**) on pre-exposure days for animals injected with cocaine or saline (vehicle).

The 2 × 5 mixed model ANOVA on body weight during pre-exposure revealed a main effect of Injection Day [F(4, 248) = 117.518, *p* < 0.001] but not of Pre-exposure Drug [F(1, 62) = 0.029, *p* = 0.865] (see Figure 5; left panel). There was no significant interaction of Injection Day × Pre-exposure Drug [F(4, 248) = 0.237, *p* = 0.917]. The 2 × 5 mixed model ANOVA on water consumption during pre-exposure indicated that there was a main effect of Injection Day [F(4, 248) = 8.395, *p* < 0.001] but not of Pre-exposure Drug [F(1, 62) = 0.007, *p* = 0.935] (see Figure 5; right panel). There was no significant interaction of Injection Day × Pre-exposure Drug [F(4, 248) = 0.803, *p* = 0.524]. For both body weight and water consumption, the main effect of Injection Day is due to both indices increasing across pre-exposure (regardless of pre-exposure condition).

##### Conditioned Taste Avoidance

**Figure 6:**
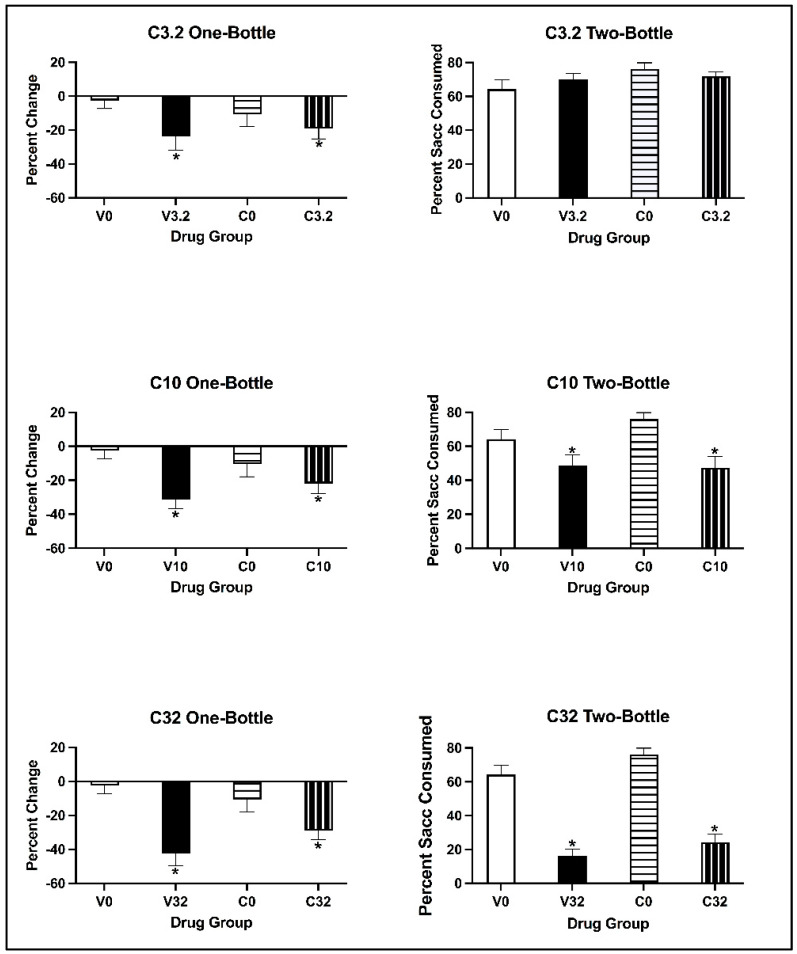
Mean (±SEM) percentage change in saccharin consumption (**left**) and percentage saccharin (±SEM) consumed (**right**) for animals pre-exposed to cocaine or vehicle and conditioned with 3.2 (**top**), 10 (**middle**) and 32 (**bottom**) mg/kg of eutylone during CTA acquisition (**left**) and on the two-bottle test (**right**). * Subjects injected with 3.2, 10 and 32 mg/kg (collapsed across pre-exposure conditions) significantly differed from controls.

For all doses, the two-way ANOVAs on the percentage change in saccharin consumption revealed that there was a main effect of Conditioning Drug [all values of F(1, 28) > 4.890, all *p* values < 0.035], but not of Pre-exposure Drug [all values of F(1, 28) < 0.169, all *p* values > 0.684]. There was no significant interaction of Pre-exposure Drug × Conditioning Drug [all values of F(1, 28) < 2.889, *p* = 0.100]. Regardless of pre-exposure, all subjects conditioned with eutylone significantly decreased saccharin consumption relative to control subjects (*p* < 0.05) (see Figure 6; left panels).

##### Two-Bottle Test

For animals injected with the 3.2 mg/kg dose of eutylone, the two-way ANOVA on the percentage of saccharin consumed on the two-bottle test revealed no main effects of Conditioning Drug [F(1, 28) = 0.045, *p* = 0.834] or Pre-exposure Drug [F(1, 28) = 2.954, *p* = 0.097] (see Figure 6; top right panel). Further, there was no significant interaction of Pre-exposure Drug × Conditioning Drug [F(1, 28) = 1.720, *p* = 0.200]. For animals injected with 10 and 32 mg/kg eutylone (see Figure 6; middle and bottom right panels), the two-way ANOVA revealed a main effect of Conditioning Drug [all F values (1, 28) = 15.493, all *p* values < 0.001]. At the 10 mg/kg dose, there was no main effect of Pre-exposure Drug [F (1, 28) = 0.913, *p* = 0.347]; at the 32 mg/kg dose, there was a significant main effect of Pre-exposure Drug [F(1, 28) = 4.687, *p* = 0.039). There was no significant interaction of Pre-exposure Drug × Conditioning Drug [all F values (1, 28) < 1.331, all *p* values > 0.258] at either the 10 or 32 mg/kg dose. Regardless of pre-exposure, subjects conditioned with eutylone consumed a significantly lower percentage of saccharin than the controls (*p* < 0.05).

##### Conditioned Place Preference

**Figure 7:**
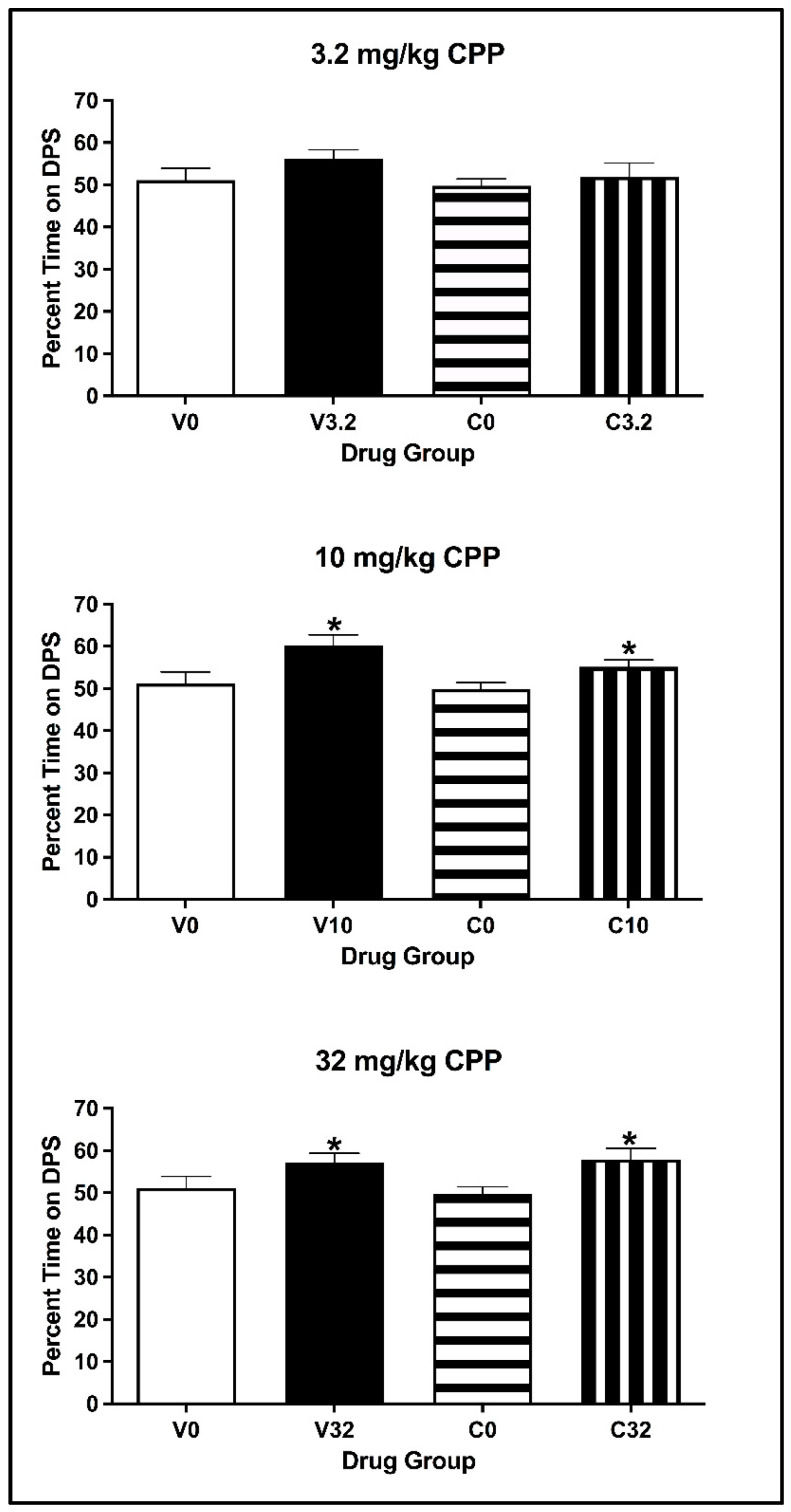
Mean (±SEM) percentage of time spent on the drug-paired side (DPS) for mice pre-exposed to cocaine or vehicle and conditioned with vehicle or 3.2 (**top**), 10 (**middle**) or 32 (**bottom**) mg/kg of eutylone. * Subjects injected with 10 and 32 mg/kg (collapsed across pre-exposure conditions) significantly differed from vehicle.

For animals injected with 3.2 mg/kg eutylone, the two-way ANOVA on the percentage of time spent on the DPS during the CPP Post-Test revealed no main effect of Conditioning Drug [F(1, 27) = 1.916, *p* = 0.178] or Pre-exposure Drug [F(1, 27) = 1.234, *p* = 0.276] (see Figure 7; top panel). There was no significant interaction of Pre-exposure × Conditioning Drug [F(1, 27) = 0.344, *p* = 0.563]. For animals injected with 10 and 32 mg/kg, the two-way ANOVA revealed a main effect of Conditioning Drug [all F values (1, 27) > 8.576, all *p* values < 0.003], but not of Pre-exposure Drug [all F values (1, 27) < 2.043, all *p* values > 0.164] (see Figure 7; middle and bottom panels). There was no significant interaction of Pre-exposure × Conditioning Drug [all F values (1, 27) < 0.686, all *p* values > 0.415] for either the 10 or 32 mg/kg dose. Regardless of pre-exposure, subjects conditioned with eutylone spent a significantly greater percentage of time on the DPS than the controls (all *p* values < 0.05).

#### 3.2.2. MDMA Pre-Exposure

##### Body Weight and Water Consumption during Pre-Exposure

**Figure 8:**
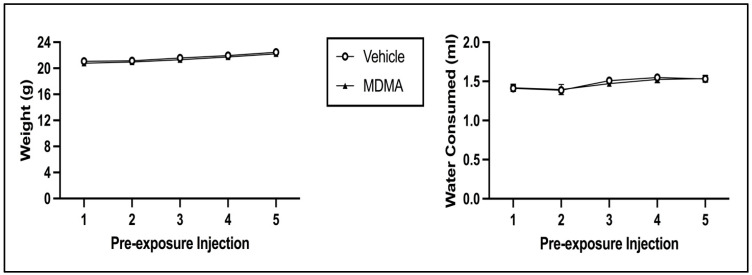
Mean (±SEM) body weight (**left**) and water consumption (**right**) on pre-exposure days for animals injected with MDMA or saline (vehicle).

The 2 × 5 mixed model ANOVA on body weight during pre-exposure revealed a main effect of Injection Day [F(4, 248) = 126.471, *p* < 0.001] but not of Pre-exposure Drug [F(1, 62) = 0.448, *p* = 0.506] (see Figure 8; left panel). There was no significant interaction of Injection Day × Pre-exposure Drug [F(4, 248) = 0.272, *p* = 0.896]. The 2 × 5 mixed model ANOVA on water consumption during pre-exposure indicated that there was a main effect of Injection Day [F(4, 248) = 8.533, *p* < 0.001] but not of Pre-exposure Drug [F(1, 62) = 0.035, *p* = 0.851] (see Figure 8; right panel). There was no significant interaction of Injection Day × Pre-exposure Drug [F(4, 248) = 0.212, *p* = 0.932]. For both body weight and water consumption, the main effect of Injection Day is due to both indices increasing across pre-exposure (regardless of pre-exposure condition).

##### Conditioned Taste Avoidance

**Figure 9:**
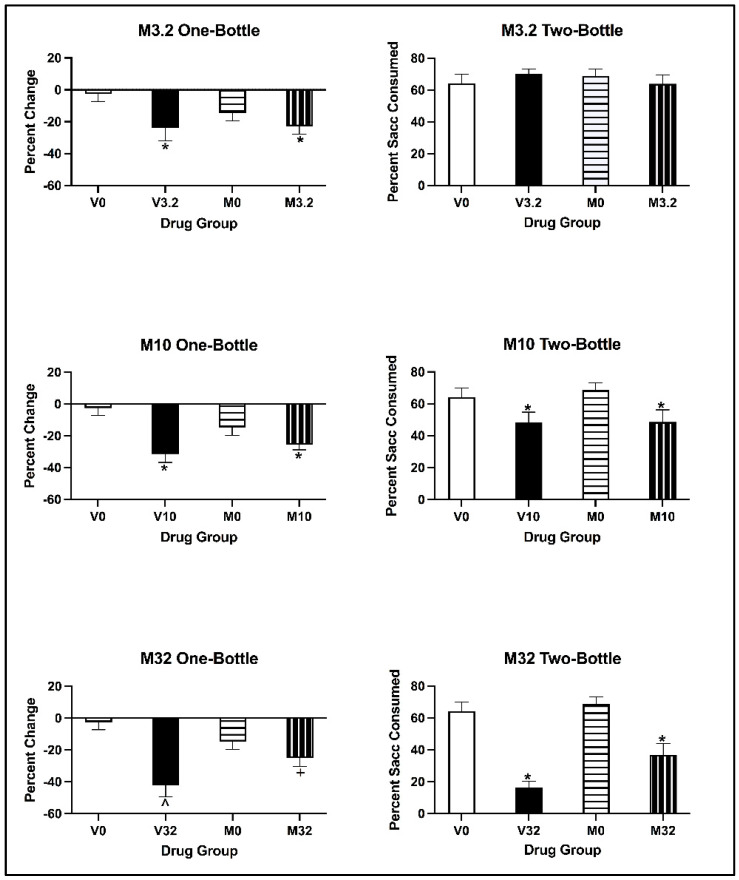
Mean (±SEM) percentage change in saccharin consumption (**left**) and percentage saccharin consumed (**right**) for animals pre-exposed to MDMA or vehicle and conditioned with vehicle or 3.2 (**top**), 10 (**middle**) or 32 (**bottom**) mg/kg of eutylone during CTA acquisition (**left**) and two-bottle test (right). * Subjects injected with 3.2 and 10 mg/kg (collapsed across pre-exposure conditions) significantly differed from vehicle. ^^^ Subjects in Group V32 significantly differed from Group V0. ^+^ Subjects in Group M32 significantly differed from Group V32.

For subjects injected with 3.2 and 10 mg/kg of eutylone, the two-way ANOVA on the percentage change in saccharin consumption revealed a main effect of Conditioning Drug [all F values (1, 28) > 6.286, all *p* values < 0.018], but not of Pre-exposure Drug [all F values (1, 28) < 0.914, all *p* values > 0.347]. There was no significant interaction of Pre-exposure Drug × Conditioning Drug for either the 3.2 or 10 mg/kg dose [all F values (1, 28) < 3.769, all *p* values > 0.062]. Regardless of pre-exposure, subjects conditioned with 3.2 and 10 mg/kg of eutylone significantly decreased saccharin consumption relative to controls (*p* < 0.05; see Figure 9, top and middle left panels). For subjects injected with 32 mg/kg of eutylone, the two-way ANOVA on the percentage change in saccharin consumption revealed a main effect of Conditioning Drug [1, 27) = 19.602, *p* < 0.001] but not of Pre-exposure Drug [F(1, 27) = 0.203, *p* = 0.656]. There was, however, a significant interaction of Pre-exposure Drug × Conditioning Drug. [F(1, 27) = 6.734, *p* = 0.015]. In relation to the two-way interaction, subjects pre-exposed to vehicle and conditioned with 32 mg/kg of eutylone (V32) significantly decreased saccharin consumption relative to controls (V0; *p* < 0.05). In contrast, subjects pre-exposed to MDMA and conditioned with 32 mg/kg (M32) did not differ from their controls (M0; *p* > 0.05). Additionally, subjects pre-exposed to vehicle and conditioned with 32 mg/kg (V32) significantly decreased saccharin consumption relative to subjects pre-exposed to MDMA (M32; *p* < 0.05; see Figure 9, bottom left panel).

##### Two-Bottle Test

For subjects injected with 3.2 mg/kg eutylone, the two-way ANOVA on the percentage of saccharin consumed on the two-bottle test revealed no main effects of Conditioning Drug [F(1, 28) = 0.012, *p* = 0.913] or Pre-exposure Drug [F(1, 28) = 0.032, *p* = 0.858] (see Figure 9; top right panel) or a significant interaction of Pre-exposure Drug × Conditioning Drug [F(1, 28) = 0.1.308, *p* = 0.263]. For subjects injected with 10 and 32 mg/kg eutyone, the two-way ANOVA on the percentage of saccharin consumed on the two-bottle test revealed a main effect of Conditioning Drug [F(1, 27) > 7.610, *p* = 0.001] but not of Pre-exposure Drug [F(1, 27) < 0.070, *p* = 0.794] (see Figure 9; middle right panel). There was no significant interaction of Pre-exposure Drug × Conditioning Drug [F(1, 27) = 0.065, *p* = 0.801]. The two-way ANOVA on the percentage of saccharin consumed on the two-bottle test revealed for subjects injected with 32 mg/kg eutylone a main effect of Conditioning Drug [F(1, 27) = 56.588, *p* < 0.001] and Pre-exposure Drug [F(1, 27) = 5.652, *p* = 0.025] (see Figure 9; bottom right panel). There was no significant interaction of Pre-exposure Drug × Conditioning Drug [F(1, 27) = 2.284, *p* = 0.142]. Regardless of pre-exposure, all subjects conditioned with eutylone consumed a significantly lower percentage of saccharin compared to the controls (*p* < 0.05).

##### Conditioned Place Preference

**Figure 10:**
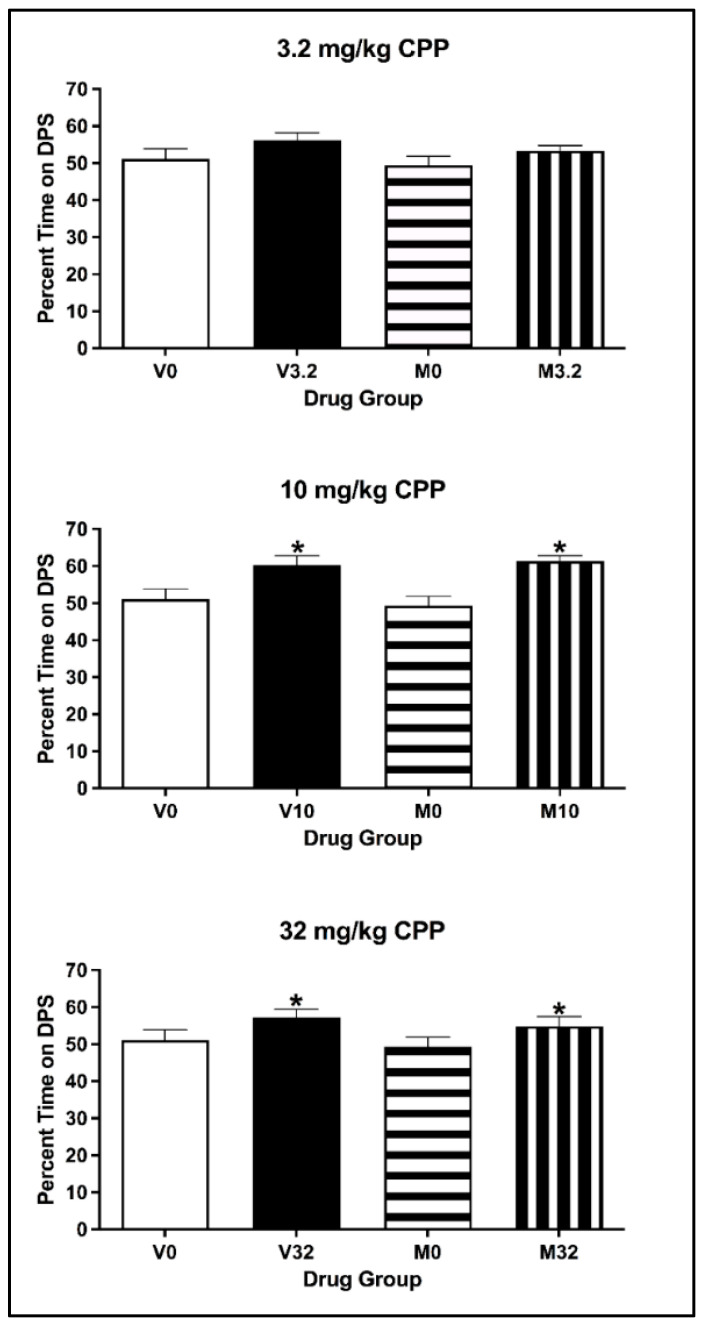
Mean (±SEM) percentage of time spent on the drug-paired side (DPS) for mice pre-exposed to MDMA or vehicle and conditioned with vehicle or 3.2 (**top**), 10 (**middle**) or 32 (**bottom**) mg/kg of eutylone. * Subjects injected with 10 and 32 mg/kg (collapsed across pre-exposure conditions) significantly differed from vehicle.

For animals injected with 3.2 mg/kg eutylone, the two-way ANOVA on the percentage of time spent on the DPS during the CPP Post-Test revealed that there was no main effect of Conditioning Drug [F(1, 26) = 3.968, *p* = 0.057] or Pre-exposure Drug [F(1, 26) = 1.062, *p* = 0.312] (see Figure 10; top panel) or any significant interaction of Pre-exposure × Conditioning Drug [F(1, 26) = 0.065, *p* = 0.801]. For animals injected with 10 and 32 mg/kg eutylone, the two-way ANOVA on the percentage of time spent revealed a main effect of Conditioning Drug [all F values (1, 27) > 4.996, all *p* values < 0.035] but not of Pre-exposure Drug [all F values (1, 27) < 0.0639, all *p* values > 0.432] (see Figure 10, middle and bottom panels). For both doses, there was no significant interaction of Pre-exposure × Conditioning Drug [all F values (1, 27) < 0.014, all *p* values > 0.552]. Regardless of pre-exposure, subjects conditioned with eutylone spent a significantly greater percentage of time on the DPS than controls (*p* < 0.05).

### 3.3. Discussion

Prior exposure to a drug can impact its aversive and rewarding effects which, in turn, may alter its acceptability and the likelihood of its intake (see [83,84]). To address this with eutylone, the effects of cocaine or MDMA pre-exposure on eutylone’s affective properties were examined in mice using a combined CTA/CPP design. Similar to the effects reported in Experiment 1, eutylone produced significant dose-dependent taste avoidance and place preference. As described, MDMA pre-exposure significantly attenuated taste avoidance induced by 32 mg/kg of eutylone, while cocaine had no effect at any dose. Neither MDMA nor cocaine pre-exposure impacted eutylone-induced place preference.

The overall limited effects of pre-exposure to cocaine and MDMA on eutylone-induced taste avoidance and place preference are surprising in the light of prior work showing significant and often robust attenuation and sensitization of such effects by drug history. For example, in one of the initial assessments of the effects of drug pre-exposure in taste avoidance learning Berman and Cannon [85] reported that rats exposed to ethanol prior to taste avoidance conditioning displayed attenuated ethanol-induced avoidance relative to non-pre-exposed subjects. Subsequent to this demonstration, exposure to a wide array of drugs has been reported to weaken taste avoidance conditioning with itself [86,87,88] and with compounds with shared neurochemical activity or effects ([55,56,89,90,91]; for reviews, see [1,2,91]). Similarly, exposure to a drug prior to place preference conditioning can sensitize the drug’s rewarding effects. For example, pre-exposure to morphine produces faster acquisition of morphine-induced place preference (at low doses of morphine) as well as significantly greater preference (at higher doses; see [92]). As with taste avoidance conditioning, such sensitization can occur when the pre-exposure and conditioning drug are similar and different (see [93,94,95,96,97]; though see [98,99]).

Although the effects of drug history have been well characterized, reports with synthetic cathinones are relatively few (for a recent review, see [13]). For example, Gregg and his colleagues [100] reported that rats pre-exposed to mephedrone (15 mg/kg; for 5 days) displayed locomotor sensitization to a subsequent injection of cocaine (15 mg/kg) when administered 10 days later. Interestingly, cocaine pre-exposure had no effect on mephedrone-induced motor activity, suggesting an asymmetrical effect of drug pre-exposure. Mephedrone pre-exposure did not sensitize methamphetamine-induced activity (for related work with methcathinone and cocaine, see [101]). More directly related to the current work, Woloshchuk et al. [102] reported that pre-exposure to MDPV (a first-generation synthetic cathinone) significantly attenuated avoidance of saccharin induced by MDPV and cocaine (but not by the emetic LiCl) with the strongest effect occurring on itself. Interestingly, Manke et al. [58] reported that pre-exposure to methylone (another first-generation synthetic cathinone) attenuated taste avoidance induced by MDPV and MDMA (MDPV > MDMA). In related work, methylone pre-exposure had no impact on avoidance induced by the SSRI fluoxetine [103]. In relation to the effects of drug history on place preference conditioning, Lopez-Arnau et al. [104] recently reported that adolescent exposure to MDPV potentiated place preference conditioning with cocaine during adulthood (as well as reinstatement of cocaine IVSA; for recent papers showing that a history of cocaine IVSA prevented the development of a high-responder phenotype for MDPV, see [105,106]). Interestingly, ethanol pre-exposure has no impact on the second-generation synthetic cathinone a-PVP’s ability to produce CPP (although it did weaken a-PVP’s aversive effects as assessed with taste avoidance conditioning) (see [82]).

In this context, the issue becomes why exposure to cocaine or MDMA had such weak (or no) effects on eutylone. As noted above, eutylone is a reuptake inhibitor at DAT and NET as well as a substrate releaser at SERT. Given its multiple neurochemical actions, it is possible that one of its subjective effects may be more salient resulting in an overshadowing or masking of its other stimulus effects. Given that MDMA (but not cocaine) did impact eutylone’s aversive effects is consistent with the possibility that its serotonergic effects are more salient than its effects on DA and NE. Such overshadowing or masking has been reported in several assessments of drug mixtures in drug discrimination learning (DDL) procedures in which animals are reinforced for responding on a specific lever following administration of individual drugs (or drug mixtures) and on a different lever following the drug vehicle. In work with drug combinations, training with a drug mixture has been reported to generalize control only to one of its components in subsequent tests, suggestive that control may have been established only to the most salient of the drugs in combination (for examples of this selective generalization or the ability of drugs to overshadow others in this design, see [107,108,109,110,111,112], though see [113]; for reports of generalization of hybrid synthetic cathinones in DDL designs, see [114,115,116]).

The dual nature of eutylone’s mechanism of action may also produce a unique configural subjective effect that does not fully generalize to drugs with single (or different) neurochemical activity. Although such assessments have not yet been made with synthetic cathinones, several studies have demonstrated such configural effects in the DDL. For example, Troisi II et al. [117] trained rats in an operant DDL procedure with a mixture of nicotine (0.3 mg/kg) and ethanol (1 g/kg) vs. saline (see also [118]). Following acquisition of the discrimination in which the mixture controlled responding, animals were assessed for the generalization of its discriminative control to the individual elements of the compound. Under these conditions, nicotine and ethanol alone substituted partially for the compound (N > E). Following these assessments, the individual elements were presented in an extinction procedure such that they were given, but animals were never reinforced for responding. In subsequent tests with the compound, responding fully recovered. From these data, Troisi II et al. [117] argued that the compound was perceived as different from the elements, functioning as a unique configural cue. Further support for this position was provided by showing that animals could learn to discriminate the compound from the elements in an explicit discrimination procedure in which the compound was reinforced while the elements were not (treated as a saline condition). As expected, animals learned this discrimination, responding only under the compound condition. Again, these data support the position that a drug mixture was not simply perceived as the combination of the individual elements, but as a unique cue that only partially overlapped with the drugs making up the compound. Accordingly, it is possible that cocaine or MDMA alone does not produce the same unique cue that is salient to the subject when eutylone is given, limiting the effects of drug pre-exposure on eutylone’s ability to induce a taste avoidance.

### 3.4. Limitations

Surprisingly, we did not see any greater taste avoidance in the choice procedure than the one-bottle design. This was unexpected given that it is generally a more sensitive index in that animals are not required to pit the avoidance of the taste against deprivation as they can avoid the taste and consume the neutral fluid. The differences reported between the one- and two-bottle designs could be due to variability in the strength of conditioning effects [119,120]. Further, this suggests that the mouse does not defend deprivation as well as the rat (for which most of the work on one- and two-bottle assessments have been made). Mice appear not to be able to suppress consumption as effectively as rats which is interesting in the light of the fact that for a host of drugs (including eutylone), mice display significant weaker taste avoidance than rats [48,66,67,68,69], suggestive that although the drug may be aversive (as one can see with higher doses), deprivation may limit their ability to suppress consumption in general. This is speculation, but such an interpretation has been given to other subject manipulations (e.g., sex difference comparisons; see [121,122]).

In the present work, the specific procedure used during place preference conditioning was driven by initial side preference. In Experiment 1, there was no significant side preference during the Pre-Test, and as such an unbiased procedure was used in which rats were randomly assigned to a specific side prior to being injected with the drug. In Experiment 2, there was a significant side preference (in Replicate 1) which dictated the use of a biased procedure in which animals were placed on their non-preferred side prior to being injected with the drug. In Replicate 2, there was no significant side preference, but to be consistent with the first replicate within the same study, a comparable biased procedure in which all animals were injected with drug on the non-preferred side was used. Importantly, despite the use of a biased vs. unbiased procedure, significant place preferences were evident across Experiments 1 and 2 and within Experiment 2, consistent with work in place preference conditioning that reports significant effects with both designs ([123,124,125,126]; for a review see [127]).

### 3.5. Conclusions

Independent of the basis for the current results, what is striking is that a history of cocaine or MDMA had weak (and selective) effects of taste avoidance learning and no effects on place preference conditioning, suggesting that the affective properties of eutylone were relatively unaffected by this history. As described above, these results parallel those from recent assessments with eutylone’s parent compound, i.e., methylone (see [58]), that is also a hybrid drug as it is a monoamine reuptake inhibitor (DA and NE > 5-HT) with release substrate activity primarily at 5-HT. It remains to be determined whether these effects reflect unique properties of hybrid compounds such as eutylone and methylone (and other synthetic cathinones with mixed actions; see also [128]) or are instead a function of the specific parameters under which they have been assessed all reported to impact the effects of drug pre-exposure, e.g., order of drug preexposure and conditioning [56,59], doses of the pre-exposure drug [92], sex [129], adolescents vs. adults [130]. Given the extensive co-use of synthetic cathinones (see above), understanding their serial (and concurrent; see [131,132,133]) interactions may be important in predicting abuse vulnerability of this class of compounds.

## Data Availability

The data presented in this study are available upon request to the corresponding author.
